# Phage Infection Restores PQS Signaling and Enhances Growth of a Pseudomonas aeruginosa *lasI* Quorum-Sensing Mutant

**DOI:** 10.1128/jb.00557-21

**Published:** 2022-04-07

**Authors:** Nina Molin Høyland-Kroghsbo, Bonnie L. Bassler

**Affiliations:** a Department of Molecular Biology, Princeton University, Princeton, New Jersey, USA; b Howard Hughes Medical Institute, Chevy Chase, Maryland, USA; University of California San Francisco

**Keywords:** quorum sensing, bacteriophage, virulence, *Pseudomonas*

## Abstract

Chemical communication between bacteria and between bacteria and the bacteriophage (phage) viruses that prey on them can shape the outcomes of phage-bacterial encounters. Quorum sensing (QS) is a bacterial cell-to-cell communication process that promotes collective undertaking of group behaviors. QS relies on the production, release, accumulation, and detection of signal molecules called autoinducers. Phages can exploit QS-mediated communication to manipulate their hosts and maximize their own survival. In the opportunistic pathogen Pseudomonas aeruginosa, the LasI/R QS system induces the RhlI/R QS system, and in opposing manners, these two systems control the QS system that relies on the autoinducer called PQS. A P. aeruginosa Δ*lasI* mutant is impaired in PQS synthesis, leading to accumulation of the precursor molecule HHQ, and HHQ suppresses growth of the P. aeruginosa Δ*lasI* strain. We show that, in response to a phage infection, the P. aeruginosa Δ*lasI* mutant reactivates QS, which, in turn, restores *pqsH* expression, enabling conversion of HHQ into PQS. Moreover, downstream QS target genes encoding virulence factors are induced. Additionally, phage-infected P. aeruginosa Δ*lasI* cells transiently exhibit superior growth compared to uninfected cells.

**IMPORTANCE** Clinical isolates of P. aeruginosa frequently harbor mutations in particular QS genes. Here, we show that infection by select temperate phages restores QS, a cell-to-cell communication mechanism in a P. aeruginosa QS mutant. Restoration of QS increases expression of genes encoding virulence factors. Thus, phage infection of select P. aeruginosa strains may increase bacterial pathogenicity, underscoring the importance of characterizing phage-host interactions in the context of bacterial mutants that are relevant in clinical settings.

## INTRODUCTION

Quorum sensing (QS) is a bacterial cell-cell communication process that enables bacteria to collectively control gene expression and orchestrate group behaviors. QS relies on the production and release of signaling molecules called autoinducers (AIs) that accumulate at high cell density and, following detection, activate signal transduction cascades (reviewed in reference 1). The opportunistic human pathogen Pseudomonas aeruginosa harbors two canonical LuxI/R type QS synthase-receptor pairs, LasI/R and RhlI/R (2, 3). LasI synthesizes the AI 3-oxo-C12-homoserine lactone (3OC12-HSL). 3OC12-HSL interacts with its partner receptor, LasR, and the complex activates expression of genes encoding virulence factors (2, 4, 5). LasR-3OC12-HSL also activates expression of genes encoding the RhlI/R QS system as well as the genes encoding the Pseudomonas quinolone signal (PQS) QS pathway (6–8). RhlI synthesizes the AI C4-homoserine lactone (C4-HSL) that, when bound by RhlR, launches expression of a second set of virulence genes (9). The RhlR-C4-HSL complex inhibits the PQS QS system (8). PqsA to PqsD (PqsA-D) are responsible for synthesis of 2-heptyl-4-quinolone (HHQ), which is subsequently converted by PqsH into 2-heptyl-3-hydroxy-4-quinolone, the AI called PQS (10). PQS interacts with its partner receptor, PqsR, and the complex controls downstream gene expression (11).

Bacteriophages (phages) are viruses that infect bacteria. Temperate phages can undergo either lytic development, in which, following infection, they replicate and lyse the host, or lysogenic development, in which the phage integrates into the host genome and becomes a prophage. In response to particular stress cues, prophages can be induced to enter the lytic pathway (reviewed in reference 12). Cues governing the lytic pathway generally inform the prophage about the metabolic status and viability of the host bacterium (13–15). Prophages can also “eavesdrop” on host QS signaling and launch their lytic cycles exclusively at high cell density, presumably a condition that optimizes transmission to neighboring bacterial cells (16). Additionally, phages can communicate with one another to modulate their lysis-lysogeny transitions (17, 18). Phages harboring QS genes exist in both Gram-positive and Gram-negative bacteria (19, 20), and some phages encode QS inhibitors, enabling them to modify the social interactions of their hosts (21).

We previously reported that P. aeruginosa QS, through LasI and RhlI, increases expression, activity, and adaptation capability of the clustered regularly interspaced short palindromic repeats (CRISPR) and the CRISPR-associated (Cas) phage defense components in P. aeruginosa UCBPP-PA14 (called PA14) (22). We speculated that this regulatory mechanism ensures maximal expression of phage defenses at high cell density when the risk of phage infection is high due to the proximity of many host bacteria. Phage receptors are often QS regulated, which can confound interpretation of phage-QS interactions. To circumvent this issue, in our previous study, we measured the ability of CRISPR-Cas to eliminate a foreign plasmid (22).

Depending on whether the infecting agent is a plasmid or a phage, the mechanisms underlying parasitism and the outcomes to the bacterial prey differ. Here, we extend our analyses to the effects of QS on phage infection. We focus on infection of wild-type (WT) PA14 and a PA14 Δ*lasI* QS mutant by the phage JBD44, which employs the O-antigen as its receptor, as, importantly, the O-antigen is not QS regulated (23, 24). To our surprise, phage JBD44 formed normal plaques on PA14 but, in contrast, caused overgrowth of the QS mutant. Specifically, after an initial killing phase, phage JBD44 infection enhanced the growth of the PA14 Δ*lasI* strain to a level surpassing that of the uninfected strain. Restoration of expression of *lasR* and genes in the Rhl and PQS QS pathways that function downstream of the LasI/R QS system drove the enhanced bacterial growth. Moreover, phage infection restored expression of QS-activated genes encoding virulence factors. Our discovery that a P. aeruginosa QS mutant reactivates its QS pathways in response to phage infection highlights the possibility that phage infection of such P. aeruginosa mutants in clinical settings may make them faster growing and more virulent, which could present significant challenges for phage therapy.

## RESULTS

### Phage infection enhances growth of the P. aeruginosa Δ*lasI* QS mutant.

Our goal was to investigate if and how phage infection and QS, together, affect PA14 phenotypes. For such an analysis, a phage that does not use a QS-regulated receptor for infection was required. Thus, we exploited phage JBD44, which uses the non-QS-regulated O-antigen as its receptor (23, 24), so it exhibits equal infectivity irrespective of host cell QS status.

**FIG 1:**
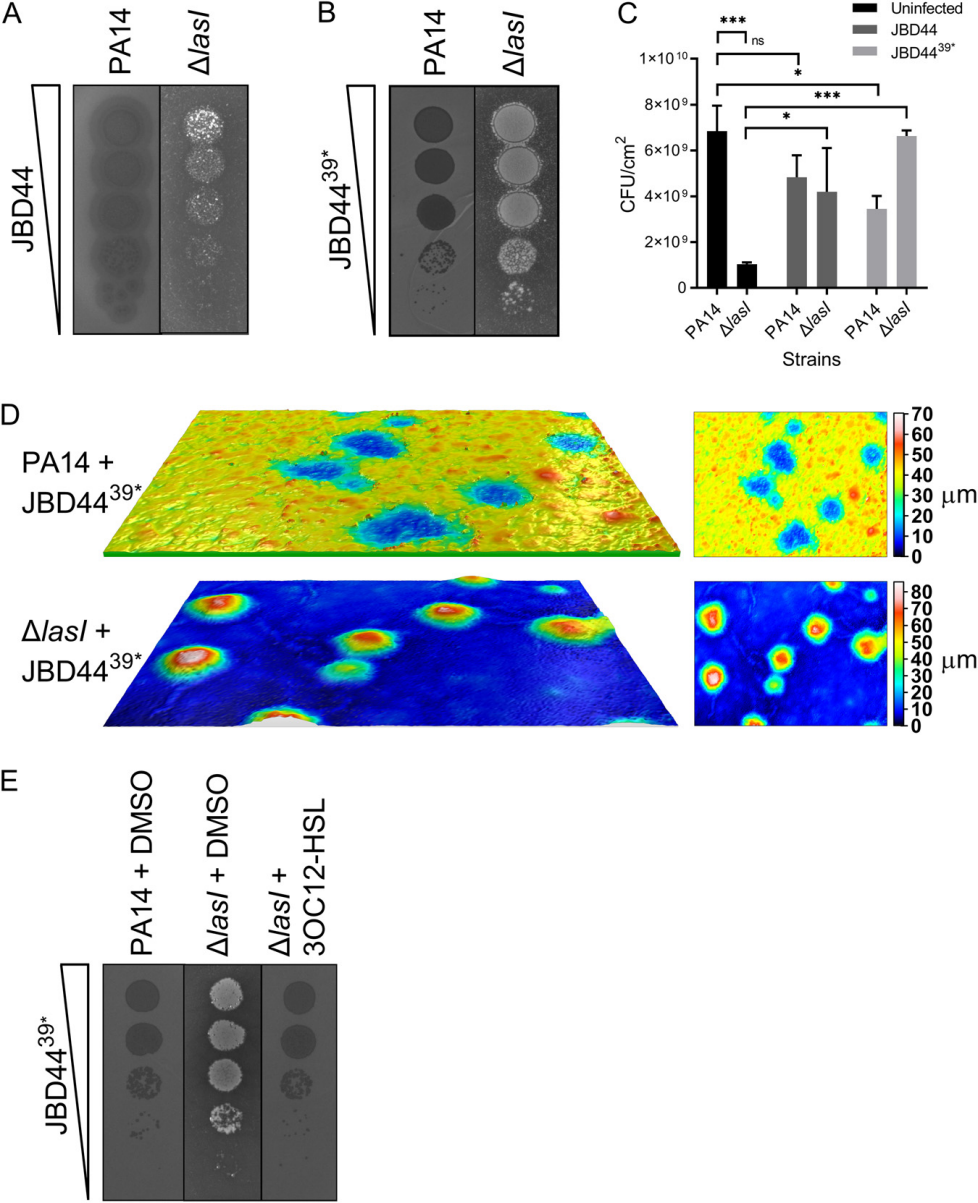
Phage infection causes enhanced growth of the PA14 Δ*lasI* mutant. Plaque assay showing 10-fold serial dilutions of phages JBD44 (A) and JBD44^39^* (B) spotted onto lawns of PA14 and the PA14 Δ*lasI* mutant. (C) Number of CFU/cm^2^ in lawns of PA14 and the PA14 Δ*lasI* mutant and in plaques following infection by the JBD44 and pJBD44^39^* phages. Error bars designate standard deviations from *n *= 3 biological replicates. ns, not significant; *, *P* < 0.05; ***, *P* < 0.001. (D) Surface profiles of individual plaques from panel B, measured using a Leica DCM 3D Micro-optical System. The images on the left show side views of plaques on lawns of PA14 (upper) and the PA14 Δ*lasI* mutant (lower). The images on the right show top views of the same plaques. Scale bars show the heights (μm) of the lawns and plaques, with black/blue representing the lowest points and red/white representing the highest points. (E) Plaque assay showing 10-fold serial dilutions of phage JBD44^39^* spotted onto lawns of PA14 and the PA14 Δ*lasI* mutant treated with DMSO (control solvent) or 2 μM 3OC12-HSL.

We assessed the plaque morphology of phage JBD44-infected PA14. JBD44 is a temperate phage, so it can enact the lytic program or it can integrate into the host chromosome. The latter process generates lysogenic cells, which are immune to killing by the same and closely related phages. Consequently, infection by phage JBD44 results in turbid plaques on lawns of PA14 (Fig. 1A). We isolated a spontaneous small-clear-plaque mutant of phage JBD44 (Fig. 1B). Sequencing revealed that the mutant phage possesses an Asn-to-Lys alteration at residue 289 (N289K) in Gp39. We call the isolated mutant phage JBD44^39^*. Gp39 is a putative tail fiber protein. Some phage tails possess depolymerase activity against bacterial capsules, and this capability drives halo formation around plaques (25). Indeed, WT phage JBD44 makes plaques with halos and the mutant JBD44^39^* phage does not (Fig. 1A and B, respectively). We suspect that the defect in lipopolysaccharide (LPS) depolymerase activity reduces adsorption, which results in phage JBD44^39^* making small plaques (26). Propagation of phage JBD44^39^* on PA14 led to rare spontaneous revertants of JBD44^39^* that restored the WT large turbid plaque morphology, including the surrounding halo. We sequenced one phage revertant. It encoded a Gp39 K289T substitution, suggesting that position 289 in Gp39 plays a central role in driving plaque size, turbidity, and halo formation.

QS regulates multiple phage defenses (22, 27–30). Thus, we investigated whether the JBD44 and JBD44^39^* phages had identical plaquing efficiencies and plaque morphologies on PA14 and on a QS mutant. To explore this question, we chose the PA14 Δ*lasI* QS mutant because the LasI synthase functions at the top of the QS hierarchy and, thus, the LasI-synthesized 3OC12-HSL AI launches the RhlI/R and PQS QS cascades. As described above, both phages formed typical plaques on lawns of PA14 (Fig. 1A and B). In contrast, on lawns of the PA14 Δ*lasI* strain, both phages promoted bacterial growth, but growth was most pronounced with the phage JBD44^39^* mutant (Fig. 1A to C). On the PA14 Δ*lasI* strain, both phages caused enhanced bacterial growth such that the colonies protruded above the lawn. With more specificity, infection of PA14 with phage JBD44^39^* resulted in concave plaques with ∼35-μm depressions below the surface of the lawn (Fig. 1D). In contrast, phage JBD44^39^*-driven enhanced growth enabled the colonies to achieve a height of approximately 70 μm above the PA14 Δ*lasI* cell lawn (Fig. 1D). Normal plaque morphology was restored to the phage JBD44^39^*-infected PA14 Δ*lasI* mutant when exogenous 2 μM 3OC12-HSL was supplied (Fig. 1E). Thus, the absence of the LasI QS synthase is required for enhanced cell growth following phage infection. The PA14 Δ*lasI* strain did not show any growth enhancement following infection in liquid medium (see Fig. S1 in the supplemental material), suggesting that this response is specific to a structured environment. PA14 Δ*lasI* ΔCRISPR Δ*cas* cells also showed enhanced growth in response to infection by the temperate phages DMS3, JBD13, JBD18, and JBD25 (Fig. S2A). Introduction of the ΔCRISPR Δ*cas* deletion was necessary in this set of experiments because PA14 has CRISPR-Cas-directed immunity against some of the tested phages (see the legend to Fig. S2A). These results suggest that the enhanced growth of the PA14 Δ*lasI* strain is caused by a bacterial response to phage infection rather than a process driven specifically by phage JBD44 and phage JBD44^39^*. In contrast, a virulent mutant of phage DMS3 did not drive growth enhancement (Fig. S2B), indicating that enhanced growth is a unique response to infection by temperate phages. Despite phage JBD44^39^* making clear plaques on PA14, which often indicates that a phage is virulent, and causing enhanced growth of the PA14 Δ*lasI* strain, which would indicate otherwise, the cells in both types of plaques are lysogens. We assert this because isolates from both types of plaques are resistant to superinfection (Fig. S3A); moreover, those isolates produce phage particles with the same plaque phenotypes that JBD44^39^* produces on each strain (Fig. S3B and C). Finally, in both cases, cells within the plaques contain JBD44^39^* integrated into their chromosomes (Fig. S3D).

### Plaque formation on the PA14 Δ*lasI* strain proceeds with an initial phase of lysis followed by enhanced growth.

**FIG 2:**
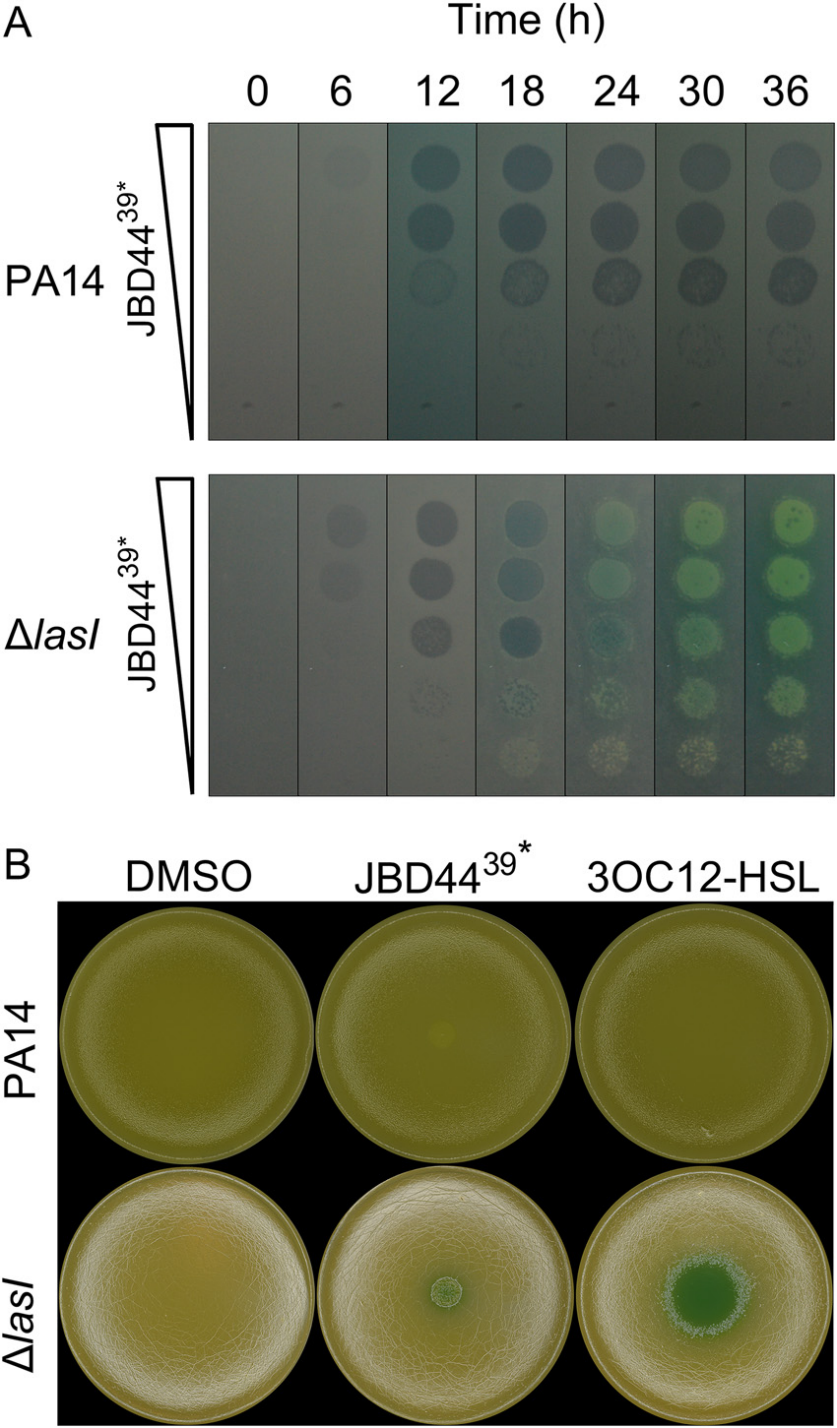
Time series and pyocyanin production profiles following phage JBD44^39^*-driven plaque development on PA14 and the PA14 Δ*lasI* strain. (A) Plaque assay showing 10-fold serial dilutions of phage JBD44^39^* spotted onto lawns of PA14 (upper) and the PA14 Δ*lasI* strain (lower). Images were acquired in the 37°C incubator. These data are also shown in Movie S1. (B) Lawns of PA14 (upper row) and the PA14 Δ*lasI* strain (lower row) to which 5 μL DMSO, phage JBD44^39^*, or 100 μM 3OC12-HSL was added in the center of each plate. Images were acquired on a photobox to accurately capture colors and iridescence. Pyocyanin appears as a blue-green colored pigment.

To investigate the development of the phage-induced growth-enhanced phenotype of the PA14 Δ*lasI* strain, we continued using the temperate phage JBD44^39^* because it induced a stronger growth enhancement than did the WT phage JBD44 (Fig. 1A to C). First, regarding timing, we imaged JBD44^39^* plaque development on lawns of PA14 and the PA14 Δ*lasI* strain every 1 h (Movie S1). Figure 2A shows that infection of PA14 caused lysis, as evidenced by turbid plaque formation on the bacterial lawns between 18 and 36 h. Similar results were obtained for the PA14 Δ*lasI* strain at initial times. At the 18-h time point, however, the plaques on the PA14 Δ*lasI* strain became increasingly turbid, indicating bacterial growth as expected from infection with a temperate phage. Growth inside the plaques continued through 30 to 36 h, by which time full overgrowth had occurred (Fig. 2A). Notably, the uninfected PA14 lawn was green due to production of the QS-regulated virulence factor pyocyanin, whereas the lawn of uninfected PA14 Δ*lasI* cells and the lawn surrounding the phage JBD44^39^*-infected PA14 Δ*lasI* cells lacked the green hue due to the absence of pyocyanin production (Fig. 2A and B). These results were expected, since LasI/R is required to activate the RhlI/R QS system, which, in turn, induces pyocyanin synthesis (3, 5, 31). Surprisingly, the surviving growth-enhanced cells in the plaques on the PA14 Δ*lasI* strain produced pyocyanin (Fig. 2A and B). Likewise, restoration of pyocyanin production occurred when PA14 Δ*lasI* cells were supplied 3OC12-HSL (Fig. 2B). Phage infection-mediated induction of pyocyanin also occurred in response to infection by the temperate phages DMS3, JBD13, JBD18, and JBD25 (Fig. S2C). Together, these results suggest that phage JBD44^39^* infection of the PA14 Δ*lasI* strain reactivates the QS pathway. Also of note, the lawn of PA14 cells surrounding where phage JBD44^39^* had been administered was smooth, whereas the lawn surrounding the infected PA14 Δ*lasI* strain showed autolysis and had an iridescent sheen (Fig. 2B). The autolytic trait was also present in colonies of the PA14 Δ*lasI* strain and in a stable PA14 Δ*lasI* JBD44^39^* lysogen (Fig. S3E).

### CRISPR-*cas* is not required for autolysis or JBD44^39^*-induced growth enhancement of the PA14 Δ*lasI* strain.

**FIG 3:**
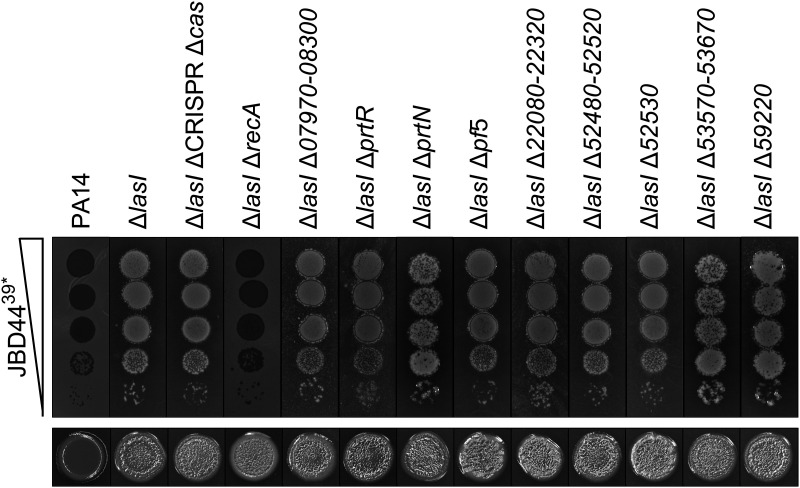
RecA is required for phage JBD44^39^*-directed growth enhancement of the PA14 Δ*lasI* mutant but not for autolysis. (Upper) Plaque assays showing 10-fold serial dilutions of phage JBD44^39^* spotted onto lawns of PA14, the PA14 Δ*lasI* strain, and the PA14 Δ*lasI* strain with the designated gene(s) also deleted. (Lower) Colony morphologies of the designated strains grown from 5 μL overnight cultures (each image is 1 cm wide).

We wondered if the autolytic trait of the PA14 Δ*lasI* strain and the ability of phage JBD44^39^* to drive enhanced cell growth were connected to the CRISPR-Cas system. Autolysis can be induced by activation of prophages residing within bacterial host genomes. Indeed, PA14 lysogenized by phage DMS3 autolyses when grown as a biofilm due to autoimmunity stemming from CRISPR-Cas-directed targeting of the DMS3 prophage in the host chromosome (32). Phage JBD44 is not inherently targeted by the PA14 CRISPR-Cas system because the PA14 CRISPR arrays do not encode CRISPR RNAs targeting this phage. However, CRISPR adaptation against phage JBD44^39^* could occur. If so, adaptation would drive subsequent targeting of phage JBD44^39^*. To test whether CRISPR-Cas is required for autolysis and for phage JBD44^39^* to drive enhanced growth of the PA14 Δ*lasI* strain, we infected a PA14 Δ*lasI* ΔCRISPR Δ*cas* mutant with phage JBD44^39^*. Figure 3 (upper) shows that there is no difference in plaque morphology between the PA14 Δ*lasI* strain and the PA14 Δ*lasI* ΔCRISPR Δ*cas* strain following phage JBD44^39^* infection, and like the PA14 Δ*lasI* strain, the PA14 Δ*lasI* ΔCRISPR Δ*cas* strain is autolytic (Fig. 3, lower). These results eliminate any role for CRISPR-Cas in formation of growth enhancement and autolysis of the PA14 Δ*lasI* strain.

### RecA is required for phage JBD44^39^*-induced growth enhancement of the PA14 Δ*lasI* strain but not for the autolysis phenotype.

We wondered whether prophages or phage remnants are activated in the PA14 Δ*lasI* strain but not in PA14 and, if so, if those genes could underpin the autolysis and growth enhancement phenotypes. RecA is required for induction of prophages. Thus, we deleted *recA* from the PA14 Δ*lasI* strain and subsequently infected it with phage JBD44^39^*. In the absence of *recA*, phage JBD44^39^* formed small clear plaques (Fig. 3, upper) that consisted of lysogens (Fig. S4), demonstrating that RecA is indeed required for growth enhancement and, moreover, suggesting that a prophage is involved in driving this phenotype. The Δ*lasI* Δ*recA* mutant was autolytic, so RecA is not required for that process (Fig. 3, lower).

To test the possibility that RecA-activated prophage components promote enhanced growth, we individually eliminated all prophages and prophage elements from the PA14 Δ*lasI* strain and assayed plaque and colony morphologies following infection by phage JBD44^39^*. Specifically, we deleted genomic region *PA14_07970-08300*, encoding R-type and F-type pyocins, and the genes *prtR* and *prtN*, encoding their repressor and activator, respectively, prophage Pf5 (*PA14_48880-49030*) (33), the cryptic prophage element *PA14_22080-22320*, the cryptic prophage operon *PA14_52480-52520*, the gene encoding its repressor *PA14_52530*, the cryptic prophage element *PA14_53570-53670*, and the S5 Pyocin (*PA14_59220*). Phage JBD44^39^*-mediated growth enhancement occurred for all of the deletion mutants, and all of the mutants remained autolytic (Fig. 3). These results show that, individually, no prophage or prophage component drive enhanced growth or autolysis. Thus, RecA is dispensable for autolysis but key for overgrowth in plaques on the PA14 Δ*lasI* strain but, at present, for unknown reasons.

### PqsA is required for phage JBD44^39^*-mediated growth enhancement of the PA14 Δ*lasI* strain.

**FIG 4:**
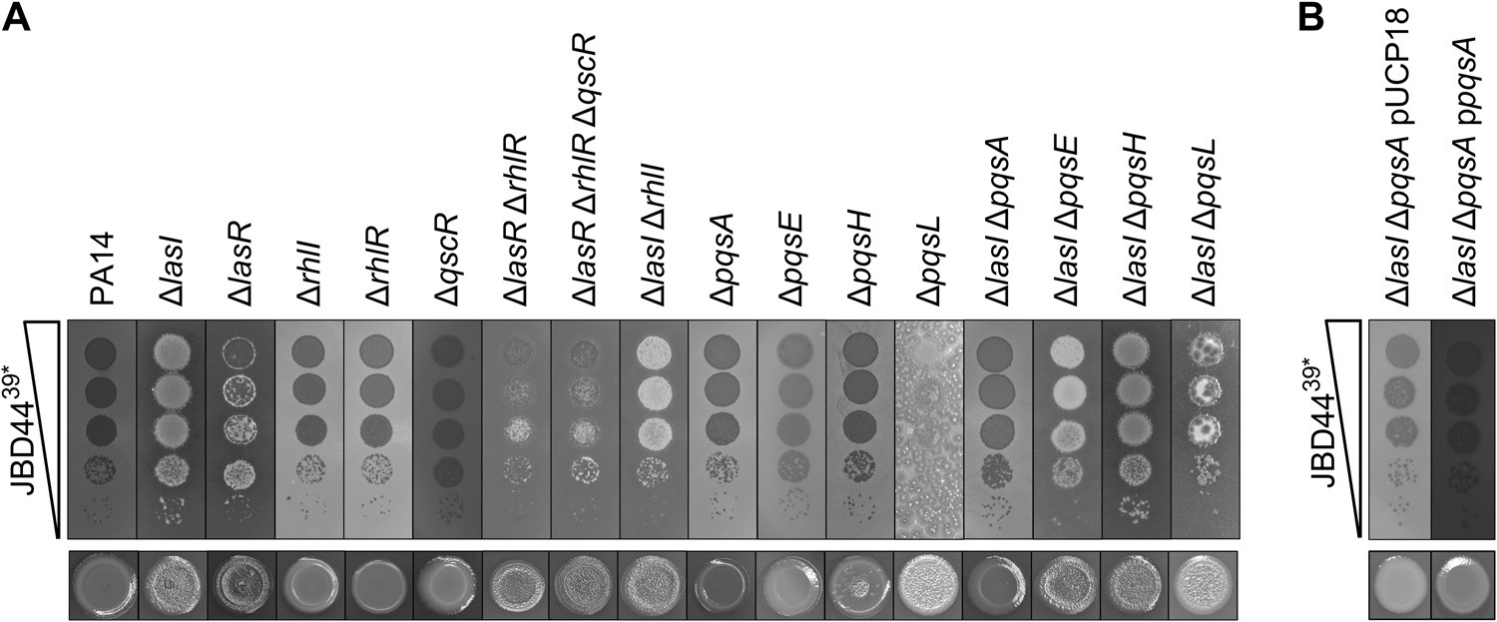
PqsA is required for phage JBD44^39^*-directed growth enhancement and autolysis of the PA14 Δ*lasI* strain. (A, upper) Tenfold serial dilutions of phage JBD44^39^* were spotted onto lawns of PA14 and the designated mutants. (Lower) Colony morphologies of the designated strains grown from 5 μL overnight cultures (each image is 1 cm wide). (B) Tenfold serial dilutions of phage JBD44^39^* were spotted onto lawns of the PA14 Δ*lasI* Δ*pqsA* strain carrying the pUCP18 vector and with *pqsA* expressed from pUCP18. Induction was with 0.2 mM IPTG.

We hypothesize that phage JBD44^39^* infection activates QS in the PA14 Δ*lasI* strain because restoration of pyocyanin production, which is a QS-controlled trait, occurred (Fig. 2A and B). To explore this notion further, and to define which QS components are involved, we assayed the plaque morphologies of phage JBD44^39^*-infected PA14 deletion mutants in the Las, Rhl, and PQS QS pathway genes and in the gene encoding the orphan QS receptor QscR (34). Figure 4A (upper) shows that, as in Fig. 1, phage JBD44^39^* formed clear plaques on lawns of PA14 and enhanced growth occurred for the PA14 Δ*lasI* strain. Clear plaques formed on several of the QS mutant strains (Fig. 4A, upper). In contrast, all the PA14 strains lacking *lasR* showed growth enhancement that was less pronounced than that for the PA14 Δ*lasI* strain (Fig. 4A, upper). Usually, a mutant lacking a QS synthase has a phenotype identical to that of the mutant lacking the cognate receptor, because AIs and partner receptors function as obligate pairs (reviewed in reference 1). However, there are exceptions in P. aeruginosa, potentially explaining the differences in plaque morphologies between the PA14 Δ*lasI* and the PA14 Δ*lasR* strains (22, 35, 36). Growth enhancement also occurred in all strains lacking *lasI*, except for the PA14 Δ*lasI* Δ*pqsA* strain, in which phage JBD44^39^* plaques were small and clear, similar to those formed on PA14 (Fig. 4A, upper). The plaques on the PA14 Δ*lasI* Δ*pqsA* strain harbored lysogenized cells (Fig. S4). Importantly, as mentioned above, PqsA is required for production of the toxic molecule called HHQ (10).

In every QS mutant strain we tested, the ability of phage JBD44^39^* to drive overgrowth correlated with a pronounced autolytic phenotype, exhibited by rough and iridescent colony surfaces (Fig. 4A, lower), suggesting that the iridescent surface precipitate could be required for phage infection-mediated growth enhancement. We note that the Δ*pqsH* mutant that is incapable of converting HHQ into PQS had partial autolysis and iridescence phenotypes, and the Δ*pqsL* mutant that cannot convert the precursor of HHQ, 2-aminobenzoylacetate, into HQNO (37, 38) exhibited an autolytic phenotype, as has been shown previously (39). Although HQNO can cause autolysis (40), deleting *pqsL* from the Δ*lasI* mutant did not relieve the autolytic phenotype, suggesting that HQNO is not causing autolysis in the Δ*lasI* strain. Rather, the precipitate is likely HHQ because deletion of *pqsA*, which eliminates HHQ production, relieves autolysis in the Δ*lasI* strain. We complemented the PA14 Δ*lasI* Δ*pqsA* strain with *pqsA* expressed from the pUCP18 plasmid; however, the phage JBD44^39^*-induced growth-enhanced and autolysis phenotypes were not restored (Fig. 4B). We do not understand this result. It is possible that our in-frame *pqsA* deletion has a polar effect on the downstream *pqsBCDE* genes, reducing their expression and lowering HHQ accumulation. We conclude that the PA14 Δ*lasI* mutant exhibits autolysis, likely due to HHQ accumulation. Phage JBD44^39^* infection relieves autolysis, thereby driving growth enhancement in the PA14 Δ*lasI* strain.

### Phage JBD44^39^* infection restores transcription of QS pathway genes and PQS production in the PA14 Δ*lasI* mutant.

**FIG 5:**
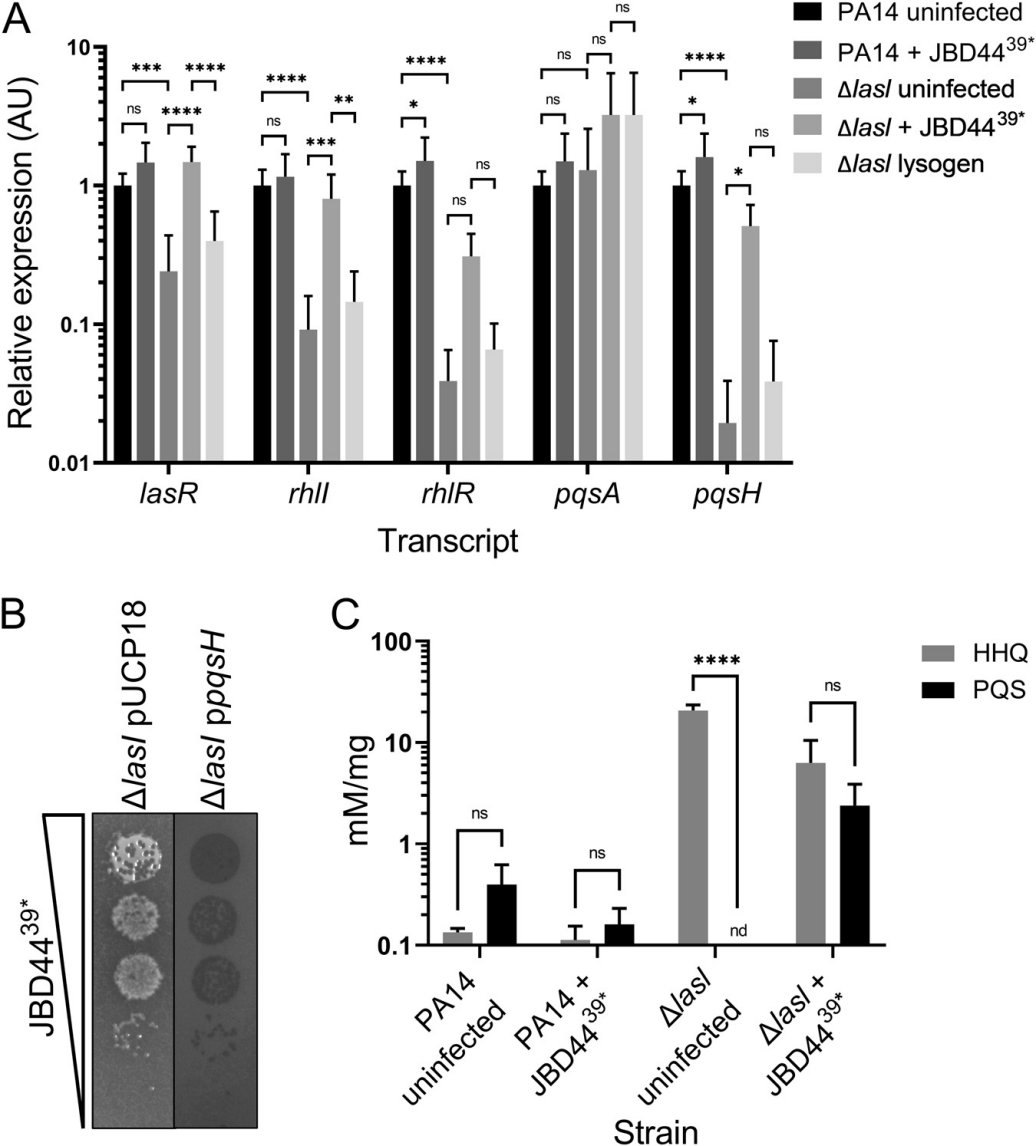
Phage JBD44^39^* infection activates expression of QS pathway genes and restores PQS production to the PA14 Δ*lasI* mutant. (A) Shown are transcript levels of the designated genes in uninfected and phage JBD44^39^*-infected lawns of PA14, the PA14 Δ*lasI* strain, and a PA14 Δ*lasI* JBD44^39^* lysogen. Relative transcript levels were normalized to 5S RNA. Error bars designate standard deviations from *n *= 9 biological replicates. ns, not significant; *, *P* < 0.05; **, *P* < 0.01; ***, *P* < 0.001; ****, *P* < 0.0001. (B) Tenfold serial dilutions of phage JBD44^39^* were spotted onto lawns of the PA14 Δ*lasI* strain harboring the empty pUCP18 plasmid or pUCP18 carrying *pqsH*. Induction was with 0.2 mM IPTG. (C) Relative levels of HHQ and PQS present in cells from uninfected lawns and from cells in plaques formed by phage JBD44^39^* infection of PA14 and the PA14 Δ*lasI* strain. HHQ and PQS levels were measured by liquid chromatography-mass spectrometry per mg lawn or per mg plaque material, and the relative concentrations were quantified using commercial HHQ and PQS standards. One cannot compare levels across the conditions but only within each condition (uninfected lawns or plaques). Error bars represent standard deviations from *n *= 3 biological replicates. ****, *P* < 0.0001.

The requirement for *pqsA* in autolysis and in phage JBD44^39^*-driven growth enhancement of the PA14 Δ*lasI* strain (Fig. 4A), coupled with our finding that cells that overgrow in the plaques produce pyocyanin, which is normally made in response to LasI/R-mediated activation of PQS QS (Fig. 2B), suggested that phage JBD44^39^* infection activated the PQS QS system in the absence of LasI. To explore this possibility, we tested whether phage JBD44^39^* infection of the PA14 Δ*lasI* strain influenced expression of QS genes. Reverse transcription-quantitative PCR (RT-qPCR) showed that, as expected given what is known about the arrangement of QS regulatory components, the PA14 Δ*lasI* strain exhibited reduced expression of the QS pathway components *lasR*, *rhlR*, *rhlI*, and *pqsH* compared to PA14 (Fig. 5A). Phage JBD44^39^* infection caused modest increases in expression of *rhlR* and *pqsH* in PA14, while infection of the PA14 Δ*lasI* strain drove increased expression of *lasR* and *rhlI* to at least 30% of their WT levels (Fig. 5A). Expression of *rhlR* and *pqsA* did not change significantly in the PA14 Δ*lasI* strain in response to JBD44^39^* infection. As noted earlier, PqsA-D synthesize the PQS precursor molecule HHQ, which, in a final biosynthetic step, is converted into PQS by PqsH. With respect to *pqsH*, in the phage JBD44^39^*-infected PA14 Δ*lasI* mutant, expression of *pqsH* changed from nearly undetectable to 50% of the PA14 level, potentially restoring the ability of the bacterium to convert HHQ into PQS. The JBD44^39^* infection-mediated increase in expression of *lasR*, *rhlI*, and *pqsH* occurred only in plaques on the PA14 Δ*lasI* strain but not in lawns of the stable PA14 Δ*lasI* JBD44^39^* lysogen (Fig. 5A), suggesting that QS activation is a response to phage infection, phage replication, and/or the process of lysogenization but does not occur in a stable lysogen.

Our finding of potential differences in HHQ production in the phage-infected mutants indicated that the molecule responsible for the iridescent sheen on the PA14 Δ*lasI* colonies was HHQ, and, if so, HHQ could drive autolysis. Evidence for this possibility comes from earlier reports showing that P. aeruginosa *lasR* null mutants form iridescent colonies and overproduce HHQ compared to WT cells (10, 41). Indeed, the PA14 Δ*lasR* strain exhibited both a sheen and autolytic phenotype similar to those of the PA14 Δ*lasI* strain (Fig. 4A, lower). Thus, we considered whether upregulation of *pqsH* expression in the JBD44^39^*-infected PA14 Δ*lasI* strain (Fig. 5A) resulted in PqsH-mediated conversion of HHQ into PQS. We overexpressed *pqsH* from pUCP18 in the PA14 Δ*lasI* strain. PqsH eliminated autolysis, iridescent sheen production, and phage JBD44^39^*-mediated growth enhancement (Fig. 5B). These results suggest that phage infection-mediated *pqsH* expression underlies this set of phenotypes. To investigate this possibility further, we measured the relative levels of HHQ and PQS in uninfected lawns of PA14 and the PA14 Δ*lasI* strain and in phage JBD44^39^*-infected plaques of PA14 and the PA14 Δ*lasI* strain using liquid chromatography-mass spectrometry. Because the uninfected and infected strains grew to different levels (Fig. 1C), direct comparisons of compound per weight of material could not be made. To circumvent this issue, we compared the ratios of HHQ to PQS in each strain under each condition. Lawns of PA14 cells possessed nearly equal ratios of HHQ and PQS irrespective of phage infection state. In contrast, lawns of the PA14 Δ*lasI* strain possessed HHQ but had no detectable PQS (*P* < 0.0001) (Fig. 5C). Importantly, the surviving, growth-enhanced JBD44^39^*-infected PA14 Δ*lasI* cells possessed readily detectable PQS and, moreover, made roughly equal amounts of HHQ and PQS (Fig. 5C). Thus, phage JBD44^39^* infection of the PA14 Δ*lasI* strain restored the ability of the strain to convert HHQ into PQS. Furthermore, these results likely explain why deletion of *pqsA* in the PA14 Δ*lasI* strain abrogated autolysis (Fig. 4A, lower): this mutant makes no HHQ. Our data are also in agreement with the observations that a P. aeruginosa PAO1 *lasR* mutant produces PQS during late stationary-phase growth and the Candida albicans QS molecule farnesol can restore PQS production in a PA14 Δ*lasR* mutant (42, 43). Thus, under particular physiological conditions, including late stationary-phase growth, in response to fungal QS signals, and following phage infection, the PQS QS pathway can be activated independently of LasI/R QS signaling.

### Phage JBD44^39^* infection restores expression of virulence genes in the PA14 Δ*lasI* strain.

Given that transcript levels of QS regulatory genes increased following phage JBD44^39^* infection of the PA14 Δ*lasI* strain (Fig. 5A), it follows that expression of QS-regulated target genes could also be upregulated. To examine this possibility, we assayed *lasB*, *rhlA*, and *phzA* expression as readouts of QS-directed virulence activity. The Las QS system promotes expression of *lasB*, encoding the extracellular virulence factor elastase (44, 45). The rhamnolipid biosynthesis gene *rhlA* is activated by the Rhl QS system (45). PQS QS upregulates expression of the *phzA* gene involved in production of phenazines, including pyocyanin, which plays a role in biofilm development and virulence (46, 47).

**FIG 6:**
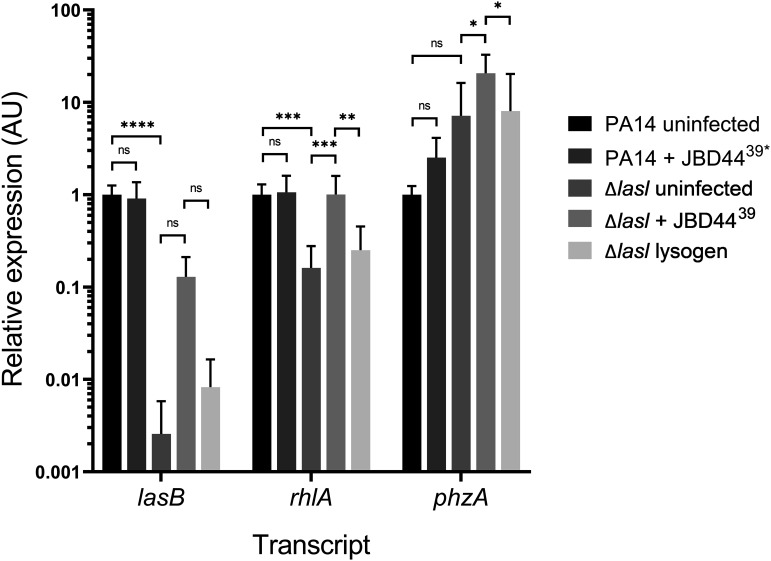
Phage JBD44^39^* infection activates expression of virulence genes in the PA14 Δ*lasI* mutant. Shown are transcript levels of the designated genes in uninfected and phage JBD44^39^*-infected PA14 and the PA14 Δ*lasI* strain and a PA14 Δ*lasI* JBD44^39^* lysogen. Relative transcript levels were normalized to 5S RNA. Error bars designate standard deviations from *n *= 9 biological replicates. ns, not significant; *, *P* < 0.05; **, *P* < 0.01; ***, *P* < 0.001; ****, *P* < 0.0001.

As expected, compared to PA14, *lasB* and *rhlA* exhibited reduced expression in the PA14 Δ*lasI* mutant (Fig. 6). In the phage JBD44^39^*-infected PA14 Δ*lasI* strain, expression of *lasB* was not changed significantly; however, *rhlA* expression was fully restored. *phzA* was also upregulated following phage JBD44^39^* infection of the PA14 Δ*lasI* strain. JBD44^39^* only drove an increase in *rhlA* and *phzA* expression following infection of the PA14 Δ*lasI* strain, as no increase occurred in the stable PA14 Δ*lasI* JBD44^39^* lysogen. Together, the data suggest that phage infection potentially can enhance select virulence traits in PA14. Notably, phage JBD44^39^* infection-mediated induction of expression of *phzA* in the PA14 Δ*lasI* strain exceeded that in the infected PA14 strain by 20-fold. *phzA* expression is activated by PQS, so this finding is consistent with the restoration of PQS production that occurs in the JBD44^39^*-infected Δ*lasI* strain (Fig. 5A and C). *phzA* encodes a pyocyanin biosynthesis pathway component, also in agreement with our observation of pyocyanin production by the JBD44^39^*-infected growth-enhanced Δ*lasI* cells (Fig. 2A and B).

## DISCUSSION

Here, we show that phage JBD44^39^* infection can increase expression of genes encoding QS regulators and downstream QS-controlled virulence genes in the P. aeruginosa PA14 Δ*lasI* strain (Fig. 5 and 6). It was previously reported that the C. albicans signaling molecule farnesol can restore QS pathways and virulence factor production in a PA14 Δ*lasR* mutant (43), demonstrating that external threats, whether from phages or fungi, can impinge on QS cascades and virulence in PA14 QS mutants.

We discovered that phage infection of the PA14 Δ*lasI* strain caused growth enhancement, exceeding that of uninfected surrounding autolytic PA14 Δ*lasI* cells, giving rise to growth-enhanced colonies that protruded above the surface of the lawn of uninfected cells (Fig. 1D). RecA was required for the growth enhancement (Fig. 3, upper) but not for autolysis (Fig. 3, lower). Endogenous prophage and phage-derived pyocin genes were individually dispensable for both phenotypes (Fig. 3), indicating that other genes downstream of RecA are involved. We found that PqsA is required for both the growth enhancement and the autolytic phenotypes exhibited by the phage-infected PA14 Δ*lasI* strain (Fig. 4A, upper and lower, respectively) as well as for the production of the PqsA-D product, the HHQ molecule, which accumulates (Fig. 4A, lower) due to decreased *pqsH* expression (Fig. 5A). Phage-mediated upregulation of *pqsH* expression in the PA14 Δ*lasI* strain (Fig. 5A) enabled conversion of HHQ to PQS (Fig. 5C), suggesting a requirement for HHQ for autolysis and phage-driven growth enhancement. This notion is supported by our findings that overexpression of *pqsH* in the PA14 Δ*lasI* strain abrogated autolysis and eliminated the enhanced growth phenotype (Fig. 5B).

We hypothesize that during initial phage JBD44^39^* infection of the PA14 Δ*lasI* strain, when QS pathways are not yet reactivated, phage infection causes normal plaque development. Over time, the PA14 Δ*lasI* cells accumulate HHQ, causing autolysis. We imagine that RecA detects the lysogenization process, and by an unknown mechanism, activated RecA drives increased *pqsH* expression. Consequently, phage JBD44^39^*-infected PA14 Δ*lasI* cells convert HHQ into PQS, which alleviates HHQ toxicity and endows infected cells with superior growth capabilities relative to the surrounding uninfected cells, which continue to suffer from toxic HHQ accumulation and, thus, autolysis.

A crucial finding of ours is that the PA14 Δ*lasI* JBD44^39^* lysogens exhibit an autolytic colony morphology identical to that of the PA14 Δ*lasI* mutant (Fig. S3E), suggesting that when the phage exists as a lysogen it does not drive sufficient upregulation of *pqsH* to restore PQS QS signaling (Fig. 5A). Rather, we imagine that active infection of nonlysogenic PA14 Δ*lasI* cells, again likely detected by RecA, triggers expression of the QS pathway components *lasI* and *rhlI*. RhlR, in turn, activates *pqsH* (43), and *pqsH* expression enables conversion of accumulated HHQ into PQS. We further hypothesize that C4-HSL released from the infected cells induces *pqsH* expression in neighboring noninfected or lysogenic cells, further contributing to conversion of HHQ into PQS. Another consequence of JBD44^39^*-mediated upregulation of QS pathway components in the PA14 Δ*lasI* strain is QS-dependent activation of expression of *rhlA* and *phzA* (Fig. 6). Increased *phzA* expression drives increased pyocyanin production. Thus, phage JBD44^39^* infection increases the virulence potential of the PA14 Δ*lasI* strain. Of note, we also observed growth enhancement and pyocyanin production in the PA14 Δ*lasI* ΔCRISPR Δ*cas* strain following infection by the temperate phages DMS3, JBD13, JBD18, and JBD25 (see Fig. S2A and C in the supplemental material), suggesting that enhanced growth, increased QS signaling, and expression of QS-regulated traits are general responses to infection by temperate phages in the PA14 Δ*lasI* strain.

Other recent findings also connect bacterial QS to interactions with phages. Bacteria use the accumulation of QS AIs as indicators of impending phage infection, and in response, they modulate their phage defenses to appropriately combat threats (22, 27–30). Phage infection of P. aeruginosa PAO1 activates expression of PQS biosynthesis genes (48) and phage infection and antibiotic-induced stress activate PA14 PQS production, which allows PQS to function as an alarm signal that alerts nearby uninfected PA14 cells to physically avoid phage-infected or antibiotic-stressed cells (49). In contrast, phage infection of Enterococcus faecalis inhibits expression of its Fsr QS system (50). Potentially, the phage used in this E. faecalis study harbors an anti-QS gene, perhaps analogous to a small protein LasR inhibitor recently discovered in the P. aeruginosa DMS3 phage (21). Moreover, phages surveil bacterial QS AIs and tune their lysis-lysogeny decisions to host cell density (16). Phages also encode their own QS systems that promote the transition from lysis to lysogeny when susceptible hosts become scarce (17). Thus, QS clearly shapes the outcomes of phage-host interactions. Because QS also regulates bacterial pathogenicity, future efforts to investigate phage-host QS interactions during bacterial infections will likely reveal additional cross-domain relationships.

Twenty-two percent of P. aeruginosa clinical isolates from cystic fibrosis patients harbor mutations in *lasR* (51). Our findings suggest that infection by temperate phages in this medical context activate P. aeruginosa QS signaling systems and thereby render bacteria that are not immediately killed by phages more virulent. The extent to which infections by temperate phages affect disease progression stemming from QS mutant P. aeruginosa strains awaits further study. Nonetheless, the results presented here imply that phage infection of such P. aeruginosa strains could worsen the outcome for patients.

## MATERIALS AND METHODS

### Bacterial strains, plasmids, and phages.

PA14 and mutants were grown at 37°C with shaking in Luria-Bertani (LB) broth or on LB agar plates solidified with 15 g agar/liter. The strains and plasmids used in this study are listed in Table S1 in the supplemental material. To construct chromosomal mutations in PA14 and the PA14 Δ*lasI* strain, DNA fragments flanking the gene(s) to be deleted were amplified by PCR, sewn together by overlap extension PCR, and cloned into pEXG2 (a generous gift from Joseph Mougous, University of Washington, Seattle, WA) using appropriate restriction sites (52). The resulting plasmids were used to transform Escherichia coli SM10λ*pir* and were subsequently mobilized into PA14 and the PA14 Δ*lasI* strain via mating. Exconjugants were selected on LB medium containing gentamicin (30 μg/mL) and irgasan (100 μg/mL), followed by recovery of mutants on M9 medium containing 5% (wt/vol) sucrose. Candidate mutants were confirmed by PCR and sequencing. *pqsH* and *pqsA* were cloned into pUCP18 using the XbaI and HindIII sites. The recombinant plasmids were maintained using carbenicillin (100 μg/mL), and gene expression was induced with 0.2 mM IPTG.

### Plaque assay.

A volume of 25 μL of an overnight culture of PA14 or a mutant strain was combined with 5 mL top LB agar (0.8% agar and 10 mM MgSO_4_ at 50°C) and plated on LB solidified with 15 g agar/liter. Phage lysates were serially diluted in SM buffer (100 mM NaCl, 8 mM MgSO_4_, 50 mM Tris HCl, pH 7.5, 0.01% gelatin) and spotted on bacterial lawns. Plates were incubated at 37°C. In Fig. 1E, the top and bottom agar were supplemented to a final concentration of 2 μM 3OC12-HSL. In Fig. 2B, concentrated 3OC12-HSL (5 μL of 100 μM 3OC12-HSL) was applied to the center of the top agar layer to achieve a high local concentration that did not diffuse into the entire plate. DMSO solvent was likewise added as the control in both cases.

### CFU count determination.

A 4-mm-diameter top agar plug was cored out of lawn or plaque material using an inverted P200 pipette tip. The plug was macerated with a piston in phosphate-buffered saline (PBS), followed by incubation at 50°C for 10 min with intermittent vortex. The suspension was serially diluted in PBS and 5 μL of each dilution was plated on LB plates, followed by overnight incubation at 37°C. CFU were enumerated and numbers of CFU/cm^2^ were calculated.

### Isolation of the phage JBD44^39^* mutant.

A spontaneous mutant of phage JBD44 that produced a small clear plaque morphology was isolated by infecting a culture of PA14 ΔCRISPR Δ*cas* with phage JBD44 and subculturing at a 1:1,000 dilution each day for 3 days. The cell-free culture fluid obtained from the overnight culture from the third passage was sterilized by vortex with 1% chloroform. This preparation was combined with 25 μL of an overnight culture of PA14 ΔCRISPR Δ*cas* strain and 5 mL of soft LB agar, overlaid on LB agar plates, and incubated at 37°C overnight. Phage JBD44 forms large turbid plaques with halos on lawns of PA14. Plaques were screened for those that were clear, indicating a spontaneous mutation favoring lysis had occurred. Plaques harboring putative mutant JBD44 phages were serially streaked three times on lawns of the PA14 ΔCRISPR Δ*cas* strain, and single plaques were isolated. The mutant phage JBD44 from these isolates was sequenced using MiSeq and analyzed relative to the phage JBD44 reference sequence NC_030929.1. A point mutation in *gp39* was identified; thus, the mutant was named JBD44^39^*.

### Microscopy.

The surface profiles of lawns of PA14 and the PA14 Δ*lasI* strain infected with phage JBD44^39^* that had been grown for 36 h were analyzed using a Leica DCM 3D Micro-optical System. A 10× objective was used with a z step size of 2 μm. A three-point flattening procedure was first performed on the agar surface to level the images. All images were generated by the LeicaMap software associated with the instrument.

### Determination of lysogenization.

Cells were isolated from six colonies that arose following phage JBD44^39^* infection of the PA14 Δ*lasI* strain. The isolates were purified by restreaking three times. Immunity against JBD44^39^* was tested using a plaque assay. Each isolate was additionally grown in LB overnight, the cells removed by centrifugation for 2 min at 12,000 rpm in a tabletop centrifuge, and the clarified supernatants were sterilized by treatment with chloroform. These preparations were assessed for the presence of phage JBD44^39^* by monitoring plaque formation on PA14 and the PA14 Δ*lasI* strain.

### Determination of the phage JBD44^39^* integration site.

DNA was isolated from the PA14 Δ*lasI* strain lysogenized by phage JBD44^39^* using the DNeasy blood and tissue kit (Qiagen), followed by treatment with 100 μg/mL RNase A (Qiagen). The Nextera kit (Illumina) was used to generate fragment libraries, and 250-bp paired-end sequencing was performed using MiSeq sequencing (Illumina). Sequencing reads were assembled using Galaxy (53) and aligned to the PA14 reference sequence NC_008463.1 using Mauve (54). Phage JBD44^39^* existed as an ∼49,000-bp insert between *PA14_39110* and *PA14_39130*, and the sequence was confirmed to match that of the phage JBD44 reference sequence NC_030929.1 apart from the mutation that we noted occurred in the *gp39* gene.

### qRT-PCR.

Bacterial lawns or plaques were harvested after 24 h of incubation at 37°C and combined with RNAprotect bacterial reagent (Qiagen). RNA was purified using NucleoSpin RNA (Macherey-Nagel) and DNase treated (RNaseOUT; Thermo Fisher). cDNA was synthesized using SuperScript IV reverse transcriptase with random primers (both from Thermo Fisher) and quantified using PerfeCTa SYBR green FastMix low ROX (Quanta Biosciences).

### Liquid chromatography-mass spectrometry detection of HHQ and PQS levels.

After 30 h of incubation at 37°C, 50 mg of bacterial lawns or plaques was harvested and homogenized in 1 mL LB broth using a piston. The samples were incubated for 10 min at room temperature and subjected to centrifugation for 2 min at 12,000 rpm. The resulting clarified supernatants were sterilized using 10-kDa centrifugal filter units (Amicon Ultra). These preparations were combined 1:1 with methanol (MeOH). HHQ and PQS (Sigma) standards were prepared in 50% MeOH. Liquid chromatography-mass spectrometry was performed to quantify the compounds using a Shimadzu HPLC system as described previously (55).

### Data analysis.

Each experiment was performed at least three times. The results are shown as means ± standard deviations. *P* values were calculated using one-way analysis of variance tests for multiple comparisons with a Tukey’s *post hoc* test for significance in GraphPad Prism. The time-lapse movie was made using ImageJ.
