# Supplementary material for: Phage Infection Restores PQS Signaling and Enhances Growth of a Pseudomonas aeruginosa lasI Quorum-Sensing Mutant
Source: J Bacteriol. 2022 Apr 7;204(5):e00557-21. doi: 10.1128/jb.00557-21 (PMC9112912; doi:10.1128/jb.00557-21)
Supplement: Supplemental file 1 — Figs S1-S4, Tables S1 and S2, and legend to Video S1. Download jb.00557-21-s0001.pdf, PDF file, 1.0 MB [file jb.00557-21-s0001.pdf]

Phage infection restores PQS signaling and enhances growth of a *Pseudomonas aeruginosa lasI* quorum-sensing mutant

Nina Molin Høyland-Kroghsbo and Bonnie L. Bassler

Supplemental Information:

Supplemental Figures S1-S4

Supplemental Tables S1-S2

Supplemental Movie S1

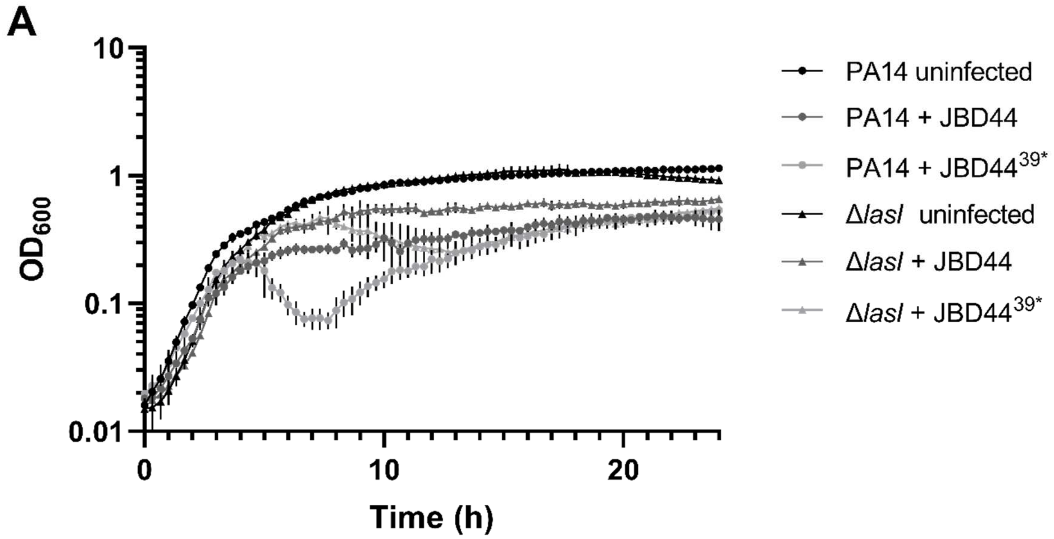

Supplemental Figure S1. Growth curves of uninfected and phage infected strains. The indicated strains were grown in liquid LB medium at 37 °C with shaking. Infections were carried out at an MOI of 1. Growth was measured as OD_{600} on a Synergy H1 multimode reader.

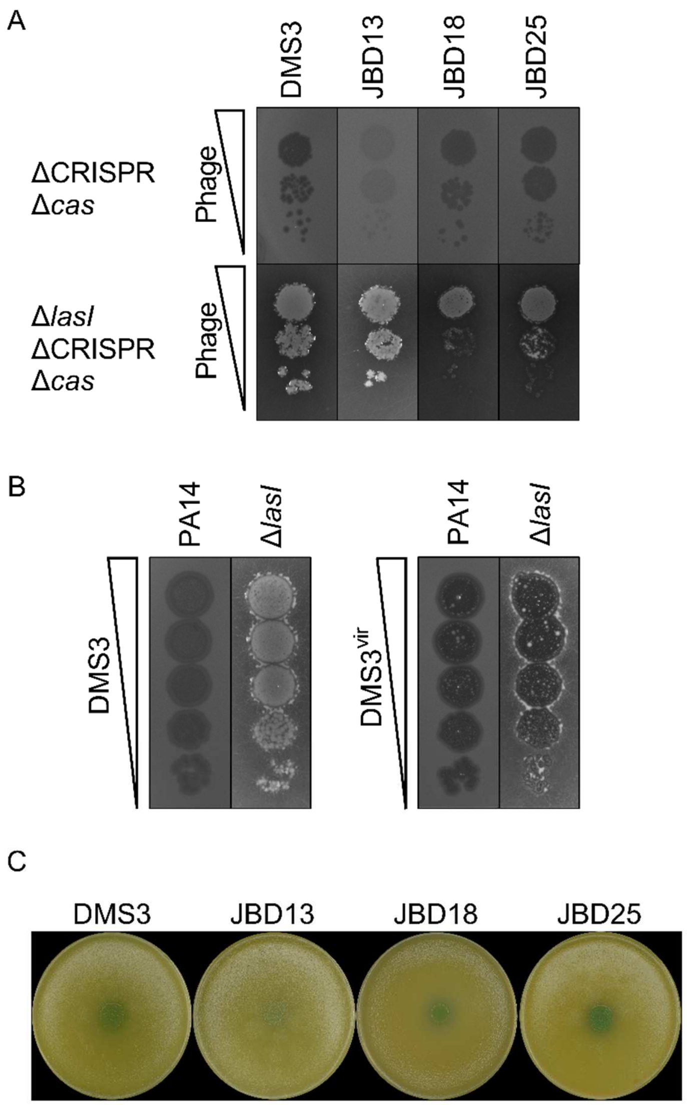

Supplemental Figure S2. Select temperate phages cause growth enhancement and restore pyocyanin production to the PA14 Δ*lasI* and PA14 Δ*lasI* ΔCRISPR Δ*cas* mutants. (A) Ten-fold serial dilutions of phages DMS3, JBD13, JBD18, and JBD25 spotted on the PA14 ΔCRISPR Δ*cas* and the PA14 Δ*lasI* ΔCRISPR Δ*cas* strains. In this panel, ΔCRISPR Δ*cas* mutants were used because PA14 has CRISPR-Cas-directed immunity against JBD18 and JBD25. (B) Ten-fold serial dilutions of phages DMS3 (left) and DMS3^{vir} (right), on PA14 and the PA14 Δ*lasI* strain. Phage resistant colonies are visible in the DMS3^{vir} plaques (right). (C) Phages DMS3, JBD13, JBD18,

and JBD25 spotted on the PA14 Δ*lasI* ΔCRISPR Δ*cas* strain, as in Figure 2B of the main text, imaged using a photobox to capture colors and irridecence. Pyocyanin appears as a blue-green colored pigment.

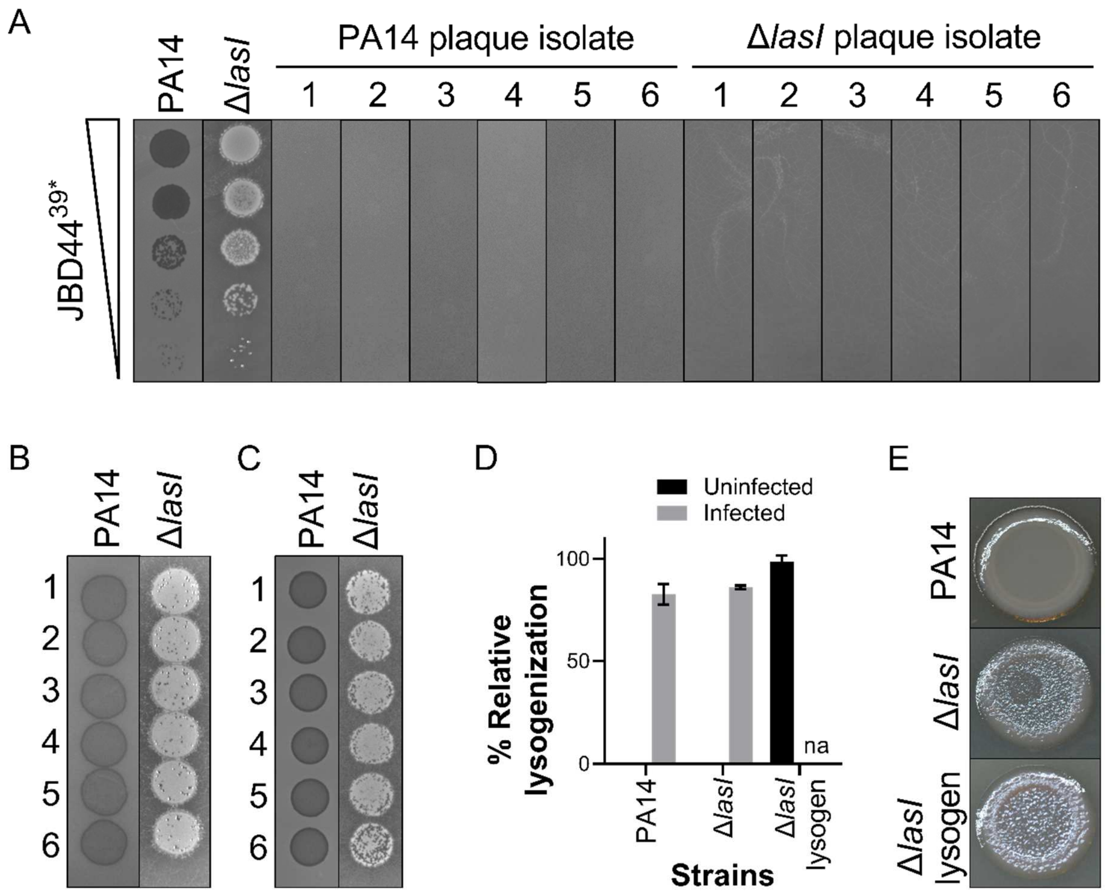

Supplemental Figure S3. Phage JBD44^{39*} lysogenizes both PA14 and the PA14 Δ*lasI* strain. (A) Plaque assay showing 10-fold serial dilutions of phage JBD44^{39*} on lawns of PA14, the PA14 Δ*lasI* strain, six isolates from PA14 that had been infected with phage JBD44^{39*}, and six isolates from the PA14 Δ*lasI* strain that had been infected with phage JBD44^{39*}. (B) Cell-free fluids prepared from overnight cultures of the six isolates obtained from plaques formed on PA14 shown in panel A spotted onto lawns of PA14 and the PA14 Δ*lasI* strain. (C) Cell-free fluids prepared from overnight cultures of the six isolates obtained from plaques formed on the PA14 Δ*lasI* strain shown in panel A spotted onto lawns of PA14 and the PA14 Δ*lasI* strain. (D) The relative percentages of cells of PA14, PA14 Δ*lasI*, and the PA14 Δ*lasI* JBD44^{39*} lysogen that were lysogenized by phage JBD44^{39*}. Black bars denote uninfected cells and gray bars

denote infected cells obtained from plaques. Relative percentages of lysogenized cells were measured by qPCR of genomic DNA using primers specific for the phage JBD44 integration site. Data are normalized to the reference gene encoding the 5S ribosomal subunit. Error bars designate standard deviations from *n* = 3 biological replicates. (E) Colony morphologies of PA14, the PA14 Δ*lasI* strain, and the PA14 Δ*lasI* strain lysogenized with phage JBD44[39*] grown from 5 µL overnight cultures (each image is 1 cm wide).

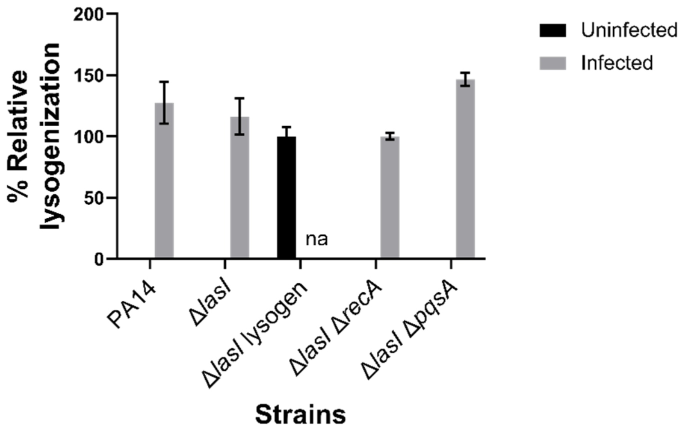

Supplemental Figure S4. Phage JBD44[39*] lysogenizes the PA14 Δ*lasI* Δ*recA* and PA14 Δ*lasI* Δ*pqsA* strains. Shown are the relative percentages of cells of the designated strains lysogenized by phage JBD44[39*]. Black denote uninfected cells and gray bars denote infected cells obtained from plaques. Error bars designate standard deviations from *n* = 3 biological replicates.

Supplemental Table S1. Bacterial strains, phages, and plasmids.

Strain, phage, or plasmid	Description	Source
<u>*P. aeruginosa*</u>		
UCBPP-PA14	Wild-type (designated PA14)	George O'Toole, Geisel School of Medicine at Dartmouth University, Hanover, NH
SM32	Δ*rhlR*	(1)
SM51	Δ*lasI*	(1)
SM52	Δ*rhlI*	(1)
SM53	Δ*lasI* Δ*rhlI*	(1)
SM67	Δ*lasR*	(1)
SM73	Δ*lasR* Δ*rhlR*	(1)
SM113	Δ*pqsA*	This study
SM563	Δ*pqsE PrhlA-mNeonGreen*	(2)
BL01	Δ*lasI* Δ*pqsE*	This study
NMHK209	Δ*lasR* Δ*rhlR* Δ*qscR*	This study
NMHK210	Δ*qscR*	This study
NMHK227	Δ*pqsH*	This study
NMHK236	Δ*lasI* Δ*pqsA*	This study
NMHK238	Δ*lasI* Δ*pqsH*	This study
NMHK272	Δ*lasI* Δ*PA14_52530*	This study
NMHK273	Δ*lasI* Δ*prtR*	This study
NMHK275	Δ*lasI* Δ*recA*	This study
NMHK287	Δ*lasI* Δ*PA14_48880-49030*	This study
NMHK288	Δ*lasI* Δ*PA14_52480-52520*	This study
NMHK292	Δ*lasI* Δ*PA14_07970-08300*	This study
NMHK316	Δ*lasI* JBD44^{39*} lysogen	This study
NMHK321	Δ*lasI* Δ*PA14_22080-22320*	This study
NMHK326	ΔCRISPR Δ*cas*	(3)
NMHK394	Δ*lasI* ΔCRISPR Δ*cas*	This study
NMHK399	Δ*lasI* Δ*prtN*	This study
NMHK420	Δ*lasI* Δ*PA14_53570-53670*	This study
NMHK421	Δ*lasI* Δ*PA14_59220*	This study
NMHK440	Δ*pqsL*	This study
NMHK441	Δ*lasI* Δ*pqsL*	This study
<u>*E. coli*</u>		
SM10λ*pir*	*thi thr leu tonA lacY supE recA*::RP4-2-Tc::Mu	Laboratory Stock

<u>Phage</u>		
JBD44	Wild-type	(4)
JBD44^{39*}	*gp39* N289K mutant	This study
JBD44^{39*rev}	Spontaneous revertant of JBD44^{39*}	This study
DMS3	Wild-type	(5)
DMS3^{vir}	Virulent mutant of DMS3	(6)
JBD13	Wild-type	(6)
JBD18	Wild-type	(6)
JBD25	Wild-type	(6)
<u>Plasmid</u>		
pEXG2	Allelic exchange vector, pBR origin, gent^R, *sacB*.	(7)
pUCP18	*Escherichia-Pseudomonas* Amp^R shuttle vector	(8)
p*pqsA*	*pqsA* driven by the P_{lac} promotor in pUCP18	This study
p*pqsH*	*pqsH* driven by the P_{lac} promotor in pUCP18	This study

Supplemental Table S2. qPCR primers used in this study.

Primer	Sequence 5'-3'	Source
5S F*	GAACCACCTGATCCCTTCCC	(1)
5S R*	TAGGAGCTTGACGATGACCT	(1)
JBD44 integration F	AGGCGTGCGTAAGTCCTCT	This study
JBD44 integration R	CAATCGATGCGAATTCTGG	This study
lasR F	ACAGCCAGGACTACGAGAAC	This study
lasR R	CCCAGAAAATCGGCAGTACG	This study
rhlI F	CCGGCATCAAGTCTTCATCG	This study
rhlI R	GTTTGCGGATGGTCGAACTG	This study
rhlR F	GAACAATTTGCTCAGCGTGC	This study
rhlR R	TTCTGGGTCAGCAACTCGAT	This study
pqsH F	ATGTCTACGCGACCCTGAAG	This study
pqsH R	AACTCCTCGAGGTCGTTGTG	This study
pqsA F	CAACACGCTCGGATTCTGTC	This study
pqsA R	AACCAGGGAAAGAACAGGCT	This study
lasB F	AGACCGAGAATGACAAAGTGGAA	This study
lasB R	GGTAGGAGACGTTGTAGACCAGTTG	This study
rhlA F	TGGCCGAACATTTCAACGT	This study
rhlA R	GATTTCCACCTCGTCGTCCTT	This study
phzA F	CTCGACCCAGAAGTGGTTCGGATCCTC	This study
phzA R	AACGGTTACAGCGGCACAGCCTGTTC	This study

*For primers, F designates forward and R designates reverse

Supplementary Movie S1. Plaque assay showing dynamics of infection of PA14 (left) and the PA14 Δ*lasI* strain (right) by phage JBD44^{39*}. Ten-fold serial dilutions of phage JBD44^{39*} were spotted on lawns of each strain. Plaque development at 37°C was imaged every 1 h. Static images from this movie are provided as the data for Fig. 2A.

Supplemental References

1. Hoyland-Kroghsbo NM, Paczkowski J, Mukherjee S, Broniewski J, Westra E, Bondy-Denomy J, Bassler BL. 2017. Quorum sensing controls the Pseudomonas aeruginosa CRISPR-Cas adaptive immune system. Proc Natl Acad Sci U S A 114:131-135.
2. Mukherjee S, Moustafa DA, Stergioula V, Smith CD, Goldberg JB, Bassler BL. 2018. The PqsE and RhlR proteins are an autoinducer synthase-receptor pair that control virulence and biofilm development in Pseudomonas aeruginosa. Proc Natl Acad Sci U S A 115:E9411-E9418.
3. Hoyland-Kroghsbo NM, Munoz KA, Bassler BL. 2018. Temperature, by Controlling Growth Rate, Regulates CRISPR-Cas Activity in Pseudomonas aeruginosa. mBio 9.
4. Phee A, Bondy-Denomy J, Kishen A, Basrani B, Azarpazhooh A, Maxwell K. 2013. Efficacy of bacteriophage treatment on Pseudomonas aeruginosa biofilms. J Endod 39:364-9.
5. Budzik JM, Rosche WA, Rietsch A, O'Toole GA. 2004. Isolation and characterization of a generalized transducing phage for Pseudomonas aeruginosa strains PAO1 and PA14. J Bacteriol 186:3270-3.
6. Cady KC, Bondy-Denomy J, Heussler GE, Davidson AR, O'Toole GA. 2012. The CRISPR/Cas adaptive immune system of Pseudomonas aeruginosa mediates resistance to naturally occurring and engineered phages. J Bacteriol 194:5728-38.
7. Rietsch A, Vallet-Gely I, Dove SL, Mekalanos JJ. 2005. ExsE, a secreted regulator of type III secretion genes in Pseudomonas aeruginosa. Proc Natl Acad Sci U S A 102:8006-11.
8. Schweizer HP. 1991. Escherichia-Pseudomonas shuttle vectors derived from pUC18/19. Gene 97:109-21.
